# Post Traumatic Stress Disorder and Substance Use Disorder as Two Pathologies Affecting Memory Reactivation: Implications for New Therapeutic Approaches

**DOI:** 10.3389/fnbeh.2019.00026

**Published:** 2019-02-13

**Authors:** Pascale Gisquet-Verrier, Claire Le Dorze

**Affiliations:** Institut des Neurosciences Paris-Saclay (Neuro-PSI), Université Paris-Sud, CNRS UMR 9197, Université Paris-Saclay, Orsay, France

**Keywords:** post-traumatic stress disorder (PTSD), memory reactivation, reconsolidation blockade, state dependency, memory integration

## Abstract

In the present review, we provide evidence indicating that although post traumatic stress disorder (PTSD) and substance use disorder (SUD) are two distinct pathologies with very different impacts on people affected by these chronic illnesses, they share numerous common characteristics, present high rates of co-morbidity, and may result from common physiological dysfunctions. We propose that these pathologies result from hyper reactivity to reminders, and thus should be considered as two disorders of memory, treated as such. We review the different possibilities to intervene on pathological memories such as extinction therapy and reconsolidation blockade. We also introduce new therapeutic avenues directly indicate by our recent proposal to replace the consolidation/reconsolidation hypothesis by the integration concept. State dependency and emotional remodeling are two innovative treatments that have already provided encouraging results. In summary, this review shows that the discovery of reactivation-dependent memory malleability has open new therapeutic avenues based on the reprocessing of pathological memories, which constitute promising approaches to treat PTSD and SUD.

## PTSD and SUD: Two Pathologies Sharing Common Characteristics

Post traumatic stress disorder (PTSD) and substance use disorders (SUDs) are two complex and specific pathologies, which, however, share many properties in common. Both are chronic and relapsing disorders, the origins of which are very well known, an aspect that is quite unusual for psychiatric disorders. These pathologies result from exposures to opposite extreme and out of the norm events, which can be schematically outlined as very negative (trauma) or very positive (drug of abuse). Only a portion of the exposed individuals (around 8%–35%; Kessler et al., 1995) are vulnerable and develop the pathology. Both disorders share some similar symptoms, including anxiety, sleep problems, hyper arousal, social isolation, and emotional numbing. They also share common risk factors, such as previous stressful life events, negative affect, having previously had another psychiatric disorder, and might be related to similar genetic susceptibility concerning the D2 receptor (Enman et al., 2015). PTSD and SUD both involve deregulations of brain reward circuitry (Schultz, 2001; Elman et al., 2005; Pierce and Kumaresan, 2006; Hopper et al., 2008) and present with very high levels of comorbidity (around 40%; Stewart et al., 1998). There is no specific treatment for these pathologies that has demonstrated its efficacy over a long period of time. Finally, another important common characteristic of these two pathologies must be emphasized: their sensitivity to cues associated with the source of the pathology i.e., the trauma or the drug. In both situations, patients tend to avoid exposure to these cues, known to elicit intrusive flashbacks of trauma in PTSD and drug craving in SUD, which may precipitate relapse of the associated pathology, even after remission or abstinence for long period of time. All these similarities provide compelling evidence emphasizing the central role that trauma and drug reminders may have for both pathologies and strengthen the hypothesis that PTSD and SUD could possibly result from common physiological dysfunctions due to exposure to extreme conditions.

## PTSD and SUD: Two Pathologies Based on Common Physiological Dysfunctions

Traumatic events and drug experiences generate some of the most enduring forms of memories, which have the salient characteristics of being easily and vividly retrieved. As a consequence, rather than stress or reward pathologies, PTSD and SUD should instead be considered as memory pathologies (Gisquet-Verrier, 2009; Gisquet-Verrier et al., 2017). There are abundant data suggesting that both may originate from a hyper reactivity to reminders. In PTSD patients, the susceptibility to trauma reminders leads to frequent re-experiencing of the trauma accompanied by vivid emotional responses, which maintain anxiety responsible for arousal, sleep disorder, social isolation, etc. and sustain the pathology over time. In SUD patients, the hyper reactivity to drug taking reminders induces frequent and intense drug craving, responsible for the urge of drug taking, accounting for chronic relapses that characterize this pathology. Interestingly, extended evidence coming from cerebral imagery indicates that exposure to trauma or drug reminders activate similar brain circuitry involving among other areas, the amygdala, ventral striatum, ventro-tegmental area, as well as the prefrontal cortex (PFC; Rauch et al., 2006; Bremner, 2007; Carrión et al., 2010; Jovanovic et al., 2013; Johnson et al., 2013; Jasinska et al., 2014; Lowen et al., 2015).

**Figure 1:**
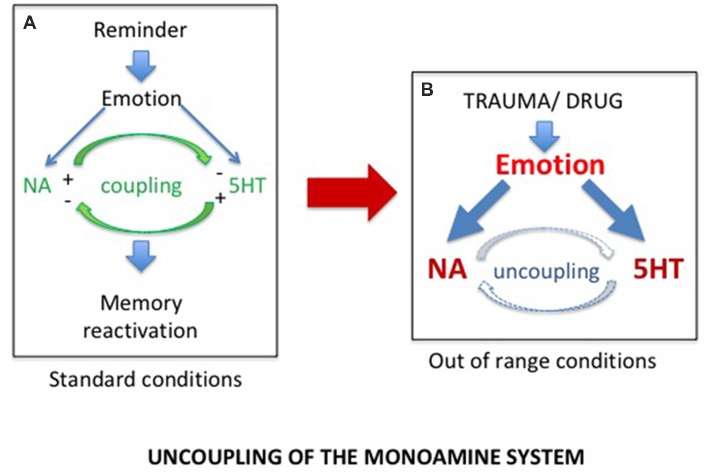
**(A)** In normal conditions, exposure to a reminder induces self-regulated noradrenergic (NA) and serotonergic (5HT) releases required for memory reactivation. **(B)** Exposure to extreme conditions, such as trauma or drug of abuse experiences, induces dramatic increases in NA and 5HT release, responsible for the uncoupling of the monoamine systems.

Accordingly, although PTSD and SUD are obviously two different pathologies with different characteristics and consequences, we proposed that they rely on common physiological processes, the disruption of which could account for a hypersensitivity to reminders restricted to the drug and trauma related memories (Gisquet-Verrier, 2009). Such a view, which could well account for the strong comorbidity between PTSD and SUD, led us to consider the findings discovered by Tassin (2008), who demonstrated that mice repeatedly exposed to various drugs of abuse exhibited large increases of noradrenaline (NA) and serotonin (5-HT) release within the prelimbic part (PL) of the medial PFC (mPFC), as well as large increases in locomotor behavior, in response to a drug activating these systems (Lanteri et al., 2008, 2014). To account for these behavioral and neurochemical sensitizations, these authors proposed that after repeated drug injections, the reciprocal control exerted by noradrenergic and serotonergic systems was disrupted, leading to an uncoupling of monoaminergic systems, accounting for their increased release (Tassin, 2008; see Figure 1). They further indicated that, through projections to the ventral tegmental area (VTA) and the nucleus accumbens (NAc), the neurochemical sensitization could be responsible for the locomotor sensitization also observed in these mice (Pierce and Kalivas, 1997; Steketee and Kalivas, 2011).

We considered these results as potential support for our views since memory reactivation has been shown to depend on the integrity of the PFC and to require activation of the noradrenergic system (Devauges and Sara, 1991; Botreau et al., 2004; Sara, 2009). It was thus important to determine whether similar results could be obtained in rodents exposed to a PTSD model. In a series of experiments, we recently explored this hypothesis using the single-prolonged stress (SPS) procedure (Liberzon et al., 1997; Lisieski et al., 2018), a PTSD model known to provide a behavioral phenotype resembling PTSD (Toledano et al., 2013; Enman et al., 2015; Le Dorze and Gisquet-Verrier, 2016a), including the fact that it only affects a subset of the exposed population (Toledano and Gisquet-Verrier, 2014; Le Dorze and Gisquet-Verrier, 2016b).

We demonstrated that, similar to mice, rats repeatedly exposed to amphetamine injections, as well as SPS vulnerable rats, developed long lasting behavioral sensitization (Toledano et al., 2013; Toledano and Gisquet-Verrier, 2014, 2016; Le Dorze and Gisquet-Verrier, 2016b). More recently, we showed that trauma vulnerable rats further exhibited a noradrenergic sensitization. Increases of NA releases in these rats were obtained, not only in response to an amphetamine injection known to stimulate the noradrenergic system, but also after a short exposure to a trauma reminder cue (Le Dorze et al., 2018), a finding previously obtained in rats repeatedly exposed to amphetamine injections (Toledano and Gisquet-Verrier, 2016). These findings strongly suggest trauma or drug experience involved similar physiological disruptions, resulting from exposure to extreme conditions. We proposed that exposures to special homeostatic challenges, such as severe trauma or drugs of abuse, intensely activate the noradrenergic and the serotonergic systems. According to Tassin, these exaggerated activations could break the inhibitory control that the noradrenergic and serotonergic neurons exert on one another in vulnerable individuals, leading to an uncoupling of monoaminergic systems (Lanteri et al., 2008). As a result, subsequent exposures to a reminder in these individuals will trigger a large increase in noradrenergic release within the PFC responsible for memory reactivation (see Figure 1). The implicit or explicit reactivation induces intrusive flashbacks of trauma in PTSD patients and intense craving followed by drug seeking in SUD patients, as well as to increased risks of relapse in both populations.

## How to Treat PTSD and SUD Considered as Memory Pathologies?

Considering that PTSD and SUD are two pathologies of memory, relying on a common physiological dysfunction, necessarily has consequences for the therapeutic approaches used to treat them. The first strategy is a direct consequence of our uncoupling hypothesis and has just began to be explored. The second group of treatments corresponds to those classically used to weaken memory, which have been adapted for therapy. We will see that despite the fact that the homology between SUD and PTSD has never actually been proposed, the way these pathologies are treated is very similar. Finally, the third approach corresponds to new treatments arising from the integration concept that we recently introduced as an alternative to the consolidation/reconsolidation view (Gisquet-Verrier and Riccio, 2018).

### Recoupling the Monoaminergic System

**Figure 2:**
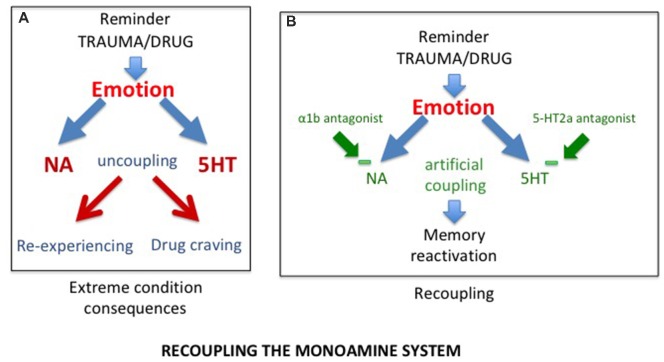
**(A)** As a result of uncoupling, exposure to trauma/drug reminders induces increased release of NA and serotonin, responsible for abnormal memory reactivations in the form of re-experiencing in post traumatic stress disorder (PTSD) and drug craving in substance use disorder (SUD) patients. **(B)** The combined action of alpha1b and 5-HT2A receptor antagonists, by reducing excessive prefrontal noradrenergic and serotonergic releases, artificially re-couples the monoaminergic systems, normalizing memory reactivation.

Considering that SUD and PTSD result from the uncoupling of the monoaminergic systems, the first approach to consider, is to correct the disruption induced by the trauma or the drugs of abuse, hence to “recouple” these systems. Indeed, it has recently been shown that delivering a combination of two blocking agents, prazosin, an antagonist of α1b-adrenergic receptors, and cyproheptadine, an antagonist of 5-HT2A receptors in alcohol dependent mice was able not only to block behavioral sensitization to amphetamine, but also to reverse their alcohol preference (Trovero et al., 2016; see Figure 2). These authors are currently investigating this approach in a clinical trial conducted on alcoholic patients. It should be noted that, when given separately, both prazozin and cyproheptadine have been shown to reduce nightmares associated with posttraumatic stress disorder (Gupta et al., 1998; Raskind et al., 2003; El-Solh, 2018). However, it will be of interest to investigate with a preclinical approach the efficacy of their combination.

### Modifying Pathological Memories

While PTSD and SUD are not generally considered to result from the same physiological dysfunction, each of them has frequently been considered to rely on abnormal learning and memory processes (Everitt, 2014; Dunbar and Taylor, 2017; Walsh et al., 2018). Accordingly, approaches consisting in decreasing the strength of these memories by extinction procedures, eliminating the pathological memories by reconsolidation blockades, or even reducing the propensity for drug/trauma associated-cues to elicit memory reactivation by emotional remodeling have been used for both PTSD and SUD.

#### Prolonged Exposure Therapy: An Extinction Procedure

**Figure 3:**
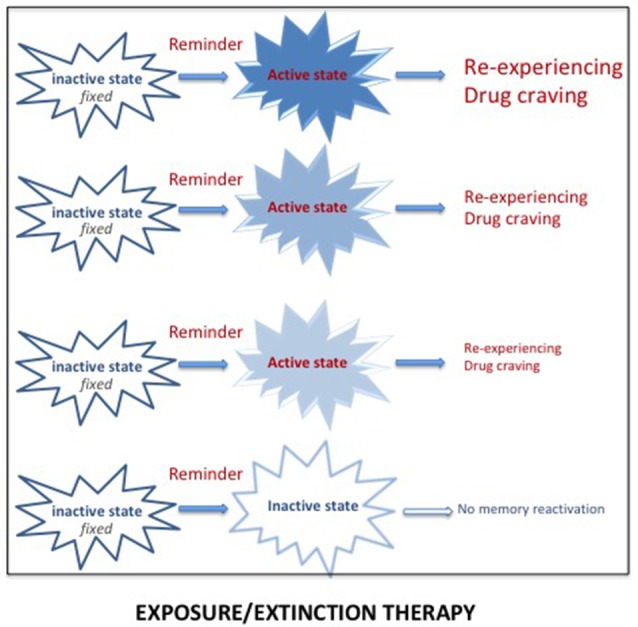
Reactivation of a pathological memory (trauma/drug) triggered by reminders (i.e., cues associated with the original memory) places the memory in an active state and induces vivid and intense remembering, taking the form of re-experiencing (for PTSD) or drug craving (for SUD). Repeated exposure to real or imaginal reminders can progressively reduce the intensity and the vividness of the memory, leading to a new memory through extinction processes. However, since this new memory competes with the original memory, spontaneous recoveries of the original memory are frequently observed.

There is extended evidence showing that exposure to reminders can evoke re-experiencing, craving and relapse. Reducing the impact of these reminders has thus been used in specific exposure therapy programs for both PTSD and SUD. Extinction corresponds to a learning process leading to progressive weakening of a learned response, due to the withdrawal of the reinforcement. Extinction serves as the basis of prolonged exposure therapy for PTSD patients, the aim of which is to reduce the emotional reactivity to trauma reminders *via* sustained imaginal and real exposure (e.g., Foa et al., 2007; see Figure 3). This form of psychotherapy, considered as an effective treatment for PTSD (Powers et al., 2010), has been extensively used, whether associated or not with a pharmacological treatment. Prolonged exposure therapy has also been considered as an effective treatment for co-occurring PTSD and SUDs (Powers et al., 2010; Mills et al., 2016). The memory retrieval-extinction procedure has also been thought of as a promising nonpharmacological method for decreasing drug craving and relapse in SUD patients. Extinction of the drug-associated cues through repeated non-reinforced presentations has also been used to diminish the impact of drug reminders on relapse to drug addiction in preclinical studies, especially when delivered in combination with d-cycloserine, a treatment known to enhance extinction (Davis, 2002; Lee et al., 2006b). A retrieval-extinction paradigm administered to heroin addicts has been shown to significantly reduce subsequent cue-induced craving (Xue et al., 2012). However, extinction learning has a number of important limitations, the most important of which is the contextual specificity of extinction learning (Bouton, 2002). Despite the undeniable success of cue exposure therapy, the long-term efficacy of this treatment is still highly questionable because extinction learning does not does not eliminate fear responses but rather creates new learning that inhibits activation of the original memory and thus is subject to relapse even after long periods of remission (Conklin and Tiffany, 2002; Foa, 2011; Myers and Carlezon, 2012).

#### Reconsolidation Blockade

The best way to treat SUD and PTSD would be to eliminate the pathological memory. Since 2000, a new approach targeting memory reconsolidation suggested that this is a possibility (Nader et al., 2000). Earlier research had demonstrated retrograde amnesia for newly acquired information, i.e., a time-dependent performance disruption induced by severe treatments such as electroconvulsive shocks, hypothermia, anesthetics or protein synthesis inhibitors. This finding led to the view that memories are not fixed immediately but undergo a consolidation period during which the memory is fragile, sustained by slow processes leading to the progressive stabilization of the memory (McGaugh, 2000). It was initially considered that once stored, however, the memory remained in that state permanently. However, numerous findings showed that memories can also be disrupted by amnesic treatments delivered shortly their reactivation/retrieval. This has been interpreted as demonstrating that reactivated memories re-enter a state of lability, and must be re-stabilized through a protein dependent process, termed reconsolidation, similar to the one engaged during the original consolidation (Nader et al., 2000; Nader and Hardt, 2009). According to that view, it was possible to disrupt remote memories, even long after their initial formation. Since that time, the opportunity to eliminate pathological memories through reconsolidation blockade has been extensively exploited in preclinical and clinical studies, for both PTSD and SUD.

**Figure 4:**
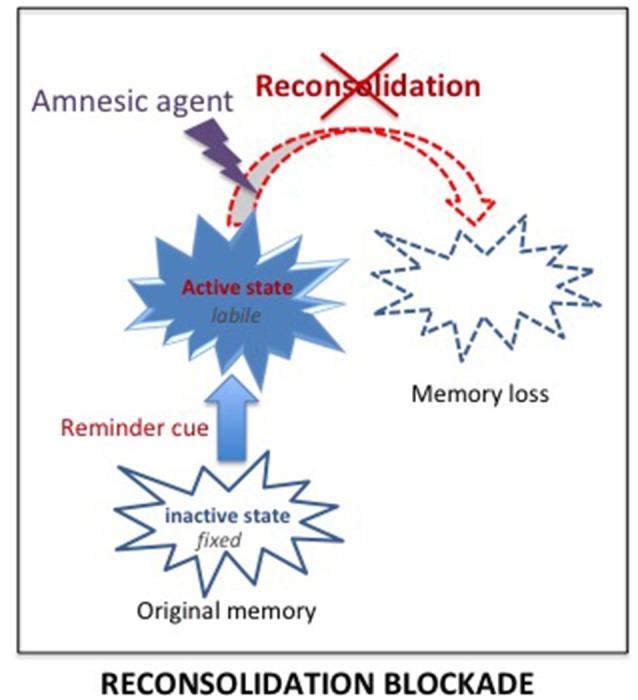
Reconsolidation blockade. According to the consolidation/reconsolidation hypothesis, reactivation destabilizes memories, re-inducing a state of lability during which they are susceptible again to amnesic treatments. Delivering an amnesic treatment at that time, is proposed to interfere with the reconsolidation processes required to re-stabilize the memory, and thus lead to a loss of the original memory.

Delivering treatments known to affect reconsolidation processes shortly after the reactivation of a memory, by preventing its re-stabilization, is intended to result in subsequent amnesia for that memory (see Figure 4). On a theoretical level, the idea has many advantages since the treatments are known to affect only the reactivated memory and not others, even closely related memories (Debiec et al., 2006).

Numerous preclinical studies have shown that treatments presumed to interfere with reconsolidation given at memory reactivation can result in a loss of the initial memory concerning either drugs of abuse (Lee et al., 2006a; Robinson and Franklin, 2007; Fricks-Gleason and Marshall, 2008) or trauma memories (Debiec and Ledoux, 2004; Muravieva and Alberini, 2010; Schneider et al., 2014). However, most of the studies, which conclusively demonstrated the possibility of inducing amnesia by the use of reconsolidation blockade, have been performed on animals, with treatments that are too toxic to be used in humans. The only one that has been tested in humans is the β-adrenoceptor antagonist, propranolol. This drug has been shown to produce retrograde amnesia when delivered shortly after training or after memory reactivation (Przybyslawski et al., 1999; Lonergan et al., 2013). As such, propranolol has been suspected of interfering with consolidation/reconsolidation processes, through mechanisms that have still not been elucidated. Particular attention has been paid to the therapeutic potential of this reconsolidation blockade in PTSD. However, despite the considerable attention to its therapeutic potential in PTSD (Lonergan et al., 2013; Brunet et al., 2018), the drug’s impact on patients is not always effective and mixed findings on propranolol and reconsolidation have been reported (Giustino et al., 2016). These findings weaken the possibility that propranolol may serve to permanently abolish trauma memories.

Preclinical studies in animal models have also demonstrated the possibility of using reconsolidation blockade to weaken or even erase drug memories (Miller and Marshall, 2005; Milton et al., 2008; Barak et al., 2013). More recently, reconsolidation blockade has been explored as a therapeutic strategy to addicted patients (Dunbar and Taylor, 2017). However, although application of reconsolidation blockade treatments has also produced mixed outcomes in SUD populations, it continues to be further investigated (Exton-McGuinness and Milton, 2018).

#### Retrieval-Dependent Approaches

Recent advances have proposed new therapeutic approaches based upon disruption of reconsolidation by behavioral interference, rather than pharmacologic blockade. It has been shown in rats and in humans that extinction learning delivered after the reactivation of a fear memory prevents the return of fear frequently obtained after extinction-based therapy (Monfils et al., 2009; Schiller et al., 2010; but see Luyten and Beckers, 2017). Similar results have been reproduced in rodent models of addiction and in human substance users (Cofresí et al., 2017; Germeroth et al., 2017). These studies have been analyzed as a demonstration that reconsolidation does not only support restabilization of memory but can also be used to update memory with new information. Such a view has been adopted in some recent studies with the aim of overwriting naturalistic maladaptive memories associated with substance use and trauma-related disorders, by the use of counterconditioning introduced after memory reactivation (Das et al., 2015; Walsh et al., 2018). Up to now, these attempts have provided interesting results but no decisive outcomes.

### New Therapeutic Approaches Provided by the Integration Concept

#### The Integration Concept as an Alternative to the Consolidation/Reconsolidation Hypothesis

**Figure 5:**
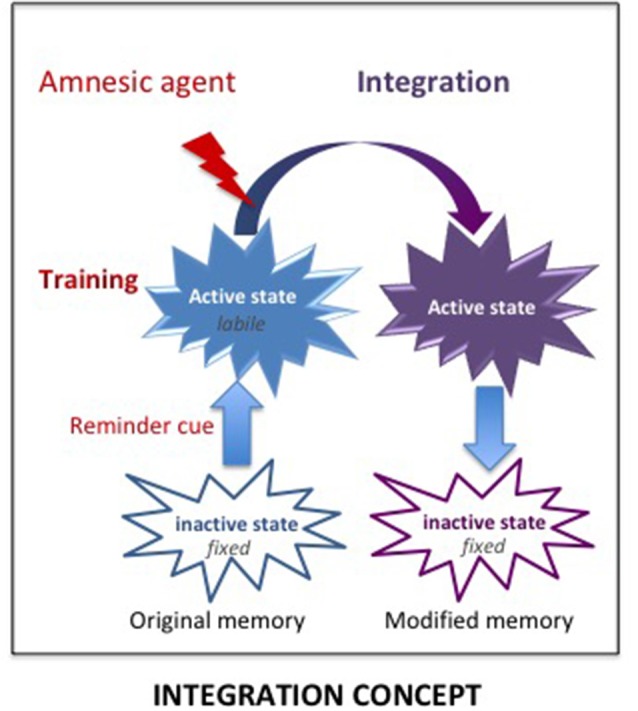
The Integration concept. According to the integration concept, the amnesic treatment delivered shortly after training induces a new internal state, which is integrated within the original memory that has been made malleable by the reminder-induced reactivation. As a result, the memory now includes the internal state provided by the amnesic agent. The lack of this determinant cue at the time of retrieval can disrupt the retrieval efficacy, leading to an apparent loss of memory, which is based on retrieval impairment.

However, the consolidation hypothesis is unable to account for some results and especially, why the “amnesia,” resulting from treatments supposed to prevent a normal functioning of the consolidation/reconsolidation processes, can be abolished either spontaneously or by pretest procedures such as delivering a reminder which can be a contextual cue, the reinforcer, and even the amnesic treatment itself (see Gisquet-Verrier and Riccio, 2018). These results demonstrate that retrograde amnesia is not due to an encoding disruption but to retrieval difficulties, a view proposed a long time ago (Miller and Springer, 1973; Miller and Matzel, 2006) but never fully considered by others. We proposed an alternative view, the integration concept, which is able to account for the variety of results obtained. According to our view, active memories (a state obtained shortly after training or memory reactivation; Lewis, 1979) are not fragile (i.e., cannot be erased) but they are malleable (i.e., can be modified) and can therefore integrate new information, including the new state induced by amnesic treatments (see Figure 5). Hence, the impairment detected at the time of testing is not due to a disruption of the fixation process, as proposed by the consolidation hypothesis, but results from retrieval difficulties due to the absence of a determinant cue: the internal state provided by the amnesic treatment^1^ which has been integrated into the initial memory. We have presented numerous examples provided by the literature indicating that the integration of that state within the initial memory disrupts the optimal functioning of the retrieval processes. However, the fact that re-introducing the drug state before testing abolishes retrograde amnesia, shows that the disruption results from retrieval difficulties due to the absence of that state, which became a determinant aspect (internal state) of the memory, a phenomenon known as state-dependency. Memory malleability, which is the main characteristic of active memories (Lewis, 1979; Gisquet-Verrier and Riccio, 2012), allows integration of new information, a process through which memories can be rapidly updated and modified. Depending on the information content, integration may update (new information), strengthen (supplementary information), weaken (interfering information) or even distort (false information) initial memory (Gisquet-Verrier and Riccio, 2018). Accordingly, integration of new information can induce changes of the memory content, even long after the events took place. Such a view opens the way to new therapeutic approaches for pathological memories. From a theoretical point of view, there are two different possibilities for new information delivered to reduce the impact of undesirable memories. First, by modifying the internal state of the subject during the reactivation of a remote memory, a state dependency procedure by which a memory can be made inaccessible, such as for retrograde amnesia. Second, by delivering a pharmacological treatment that reduces the emotional response before reactivating the pathological memory, a procedure termed emotional remodeling, which could allow the integration of a reduced emotional value within that memory.

#### State Dependency

**Figure 6:**
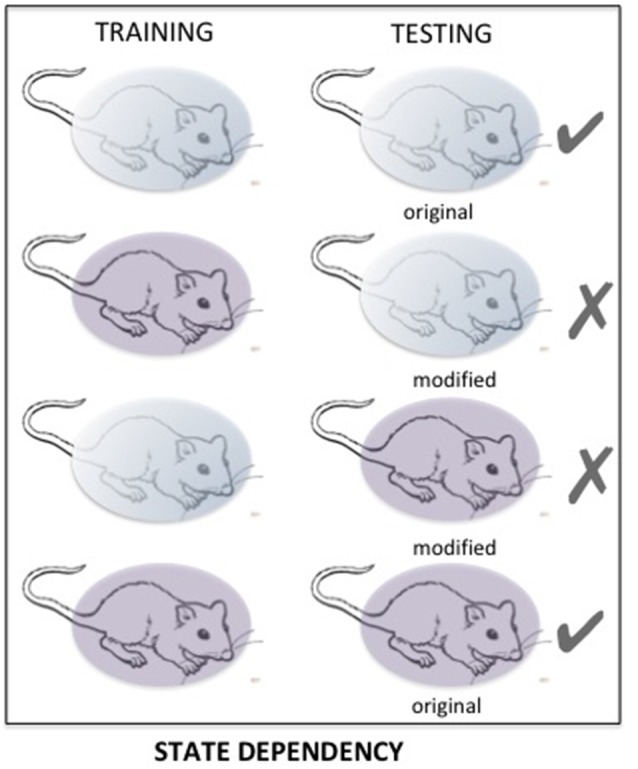
The matching of states between training and testing determines the quality of the retention performance. Changes in important aspects of that state may induce a performance disruption resulting from impaired retrieval. Here, «state» refers to internal/external contexts, which can be naturally or intentionally modified.

State-dependency is a very well-known phenomenon, accounting for retrieval difficulties occurring when the retention of information is tested in a state different from the one prevailing during the acquisition of that information (Overton et al., 1964; Koek, 2011). State dependency is a very general phenomenon, largely neglected, which is certainly a major source of retrieval variability (see Figure 6). It has been demonstrated in various circumstances with cues affecting either the internal (drug, mood) or external (environmental context) state (For a review, see Radulovic et al., 2017). Most, if not all of the psychoactive drugs such as amphetamine, cocaine, and alcohol can induce state dependency. Retrieval disruption can also be obtained by drugs which severely modifies the internal state such as lithium chloride and chemotherapy (Zarrindast et al., 2006; Gisquet-Verrier et al., 2015; Lindner et al., 2017). State dependency can also be obtained from changes concerning the surrounding environment, mood or states of consciousness (Radulovic et al., 2017). Most of the time, state dependency results in moderate memory disruptions. However, depending on the conditions, the disruption can be stronger and can even lead to complete amnesia. For instance, state dependency can explain amnesia resulting from sexual assault (“date rape”) following unintended consumption of drugs such as gamma-hydroxybutyrate (GHB; Schwartz et al., 2000; Johansson et al., 2014). State dependency has also been thought to be responsible for dissociative amnesia such as those depicted in some individuals exposed to psychological trauma (Radulovic et al., 2018). Originally, it was thought that state-dependency only resulted from an alteration of the normal state at the time when the events took place. However, recent evidence showed that state dependency can also be obtained for remote memory. Under these conditions, the remote memory must be reactivated either while the subject is under the modified state (Sierra et al., 2013), or just before introducing changes of the internal state (Gisquet-Verrier et al., 2015). The possibility of disrupting retrieval, by introducing a state dependency long after training, opens new therapeutic avenues, which have not yet been explored.

#### Emotional Remodeling: Integration of New Information

**Figure 7:**
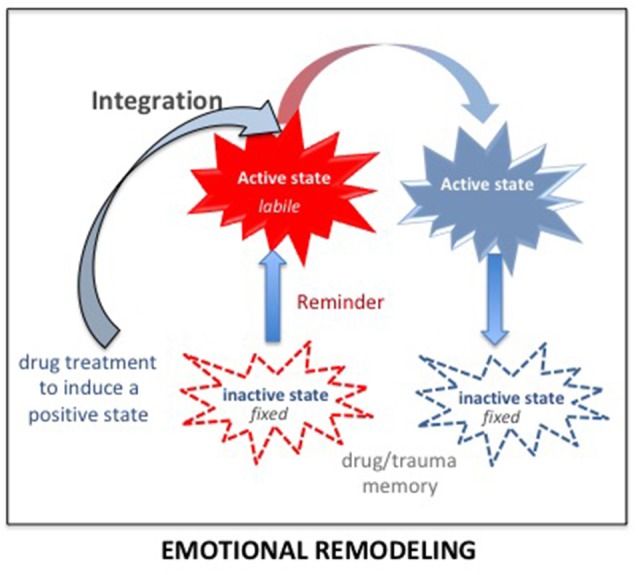
According to the integration concept, any salient information present during memory reactivation (including just before and after) is integrated within the original memory, modifying its original content. Delivering a drug inducing a positive state before the reactivation of a pathological memory (trauma/drug) strongly reduces the emotional component of the reactivated memory. Due to the malleability of the reactivated memory, this reduced emotional value will be integrated into the original memory. By doing so several times, the trauma/drug memory will progressively lose its pathologic characteristics and reach the status of an ordinary memory. This procedure has been termed: emotional remodeling.

The integration concept (Gisquet-Verrier and Riccio, 2018) provides another therapeutic approach consisting of integrating a reduced emotional component within the pathological memory. Up to now, preclinical studies showed that different types of information could be introduced while the memory is in an active state. For instance, remote memories can integrate new contextual information (Boller and Rovee-Collier, 1992; Briggs and Riccio, 2008), or a new relationship between cues (Tronel et al., 2005). There is also evidence suggesting that the emotional component of active declarative memories can be modified (Arminjon et al., 2015). Hence, we propose that preventing the occurrence of strong emotional responses elicited by the reactivation of a pathological memory, by a prior administration of a pharmacological treatment known for its relaxing properties, could allow the integration of a reduced emotional component within that pathological memory (see Figure 7). We have termed this procedure *emotional remodeling*. Interestingly, this can be achieved with propranolol, known to lower heart rate and blood pressure, but also to have anxiolytic properties (Turner and Granville-Grossman, 1965; Steenen et al., 2016). We recently delivered this β-adrenoceptor antagonist treatment to a cocaine-user patient before eliciting the reactivation of his drug memories by drug reminders. Several repeated pairings between reduced anxiety and reactivation of drug memories, associated with a cognitive behavioral therapy, have been able to reduce and then abolish drug taking and craving over a very long period of time (Chopin et al., 2016). Emotional remodeling can thus explain the effects obtained by propranolol in PTSD and SUD patient. However, according to our view, other treatments might be much more effective. In rats, using our SPS model, we showed that a single injection of d-amphetamine (known to induce a positive mood in human; Kirkpatrick et al., 2016) delivered 30 days after the trauma, durably abolished most of the SPS-induced effects. While amphetamine *per se* did not modify the behavior of non-traumatized or resilient rats, trauma susceptible rats treated with amphetamine no longer differed from controls in the symptom tests, (Toledano and Gisquet-Verrier, 2014). These results can be related to the “amphetamine narcosis,” a procedure used during the Algerian conflict in 1960, consisting of a combination of a barbiturate and amphetamine, delivered just before the reactivation of the trauma memory, which produced successful results in PTSD patients (Delay, 1949; Crocq, 1999). Amphetamine, categorized as an agonist replacement therapy, has shown efficacy in reducing cocaine intake in human addicts in multiple clinical trials (e.g., Rush and Stoops, 2012). In the 1980s, a form of amphetamine, the *3,4-methylenedioxy-methamphetamine* (MDMA), or ecstasy, a synthetic drug producing feelings of increased energy, pleasure, emotional warmth, was used as an adjunct for psychotherapy by a number of therapists in California (USA) for treatment-resistant PTSD patients (Parrott, 2007). However, MDMA-assisted psychotherapy was abandoned when the use of MDMA became illegal. Interestingly, MDMA-assisted psychotherapy has recently been re-introduced in the United States (Amoroso and Workman, 2016; Mithoefer et al., 2018). It is emphasized that the treatment, delivered together with specialized psychotherapy support, appears to facilitate the recall of traumatic memories without the patient feeling overwhelmed by the negative affect that usually accompanies such memories (Sessa, 2017). Since MDMA treatment needs to be delivered just two or three times, it is not considered likely to prime a drug dependency. These treatments have been considered to strengthen the relationship of trust between the patient and the therapist but might be rather viewed as effective drugs to induce an emotional remodeling.

Since amphetamine and MDMA are known to produce oxytocin release, a neuropeptide which increases social approach and adaptation by attenuating anxiety and stress, and globally contributes to promote “trusting behavior” (Baumgartner et al., 2008), we tested the effects of oxytocin in our previously used PTSD rodent model. In this experiment, 1 month after SPS, rats received two remodeling sessions, involving an intraventricular infusion of oxytocin, before a re-exposure to a SPS-related cue. Our results indicated that 83% of SPS-vulnerable rats treated with oxytocin showed a complete remission of PTSD-like symptoms, with no relapse up to 1 month after the treatment. In addition, we showed that oxytocin-based emotional remodeling durably reversed the neural consequences of SPS, suggesting that this treatment represents a promising approach to treat fear memory disorders (Le Dorze et al. submitted). Interestingly, the ability of oxytocin to attenuate drug seeking and craving has been recently pointed out (Sarnyai and Kovács, 2014). After drug self-administration learning in rats followed by extinction, oxytocin combined with drug-cue presentations has been reported to attenuate drug seeking during a reinstatement test. The ability of oxytocin to attenuate drug seeking and craving has been reported with various drugs of abuse, including methamphetamine, ethanol, heroin, morphine and cocaine (Sarnyai and Kovács, 2014; Leong et al., 2017).

Unlike prolonged exposure therapy, which gives rise to an extinguished memory that competes with the original memory to control behavior, emotional remodeling is supposed to modify the original memory. Hence, any new reminders encountered after the treatment will induce the reactivation of the modified memory, suggesting that the effect of emotional remodeling could be permanent.

Other procedures, presented above in the *Retrieval-dependent approach* section, such as the multisensory disgust-based counterconditioning procedure which have recently been used to re-write alcohol cue-reward associations in maladaptive reward memories (Das et al., 2015; Hon et al., 2016; Goltseker et al., 2017) could advantageously be analyzed as cases of emotional remodeling.

## Conclusion

Despite disparities based on differing theoretical backgrounds, a wealth of evidence shows that the malleability of reactivated memory has opened up new therapeutic avenues, based on the possibility of permanent modification of long-term memories. This has become an invaluable resource to find common psychotherapeutic strategies to treat pathological memories such as PTSD and SUD in the context of reactivation-dependent memory malleability. The procedure requires, first, placing patients in safe and secure conditions in order to enhance the therapeutic alliance (an effect that can be strengthened by the use of a pharmacological drug enhancing the sense of emotional well-being). Second, by exposing participants to trauma/drug reminders to reactivate the related pathological memory, in order render it malleable. The role of cognitively based therapy, frequently associated with this procedure, is to maintain the memory in an active state in the presence of new information. It must be emphasized that such a scheme not only corresponds to reconsolidation or integration-based treatments but also to others such as *eye movement desensitization and reprocessing* (EMDR), *Neuro-Linguistic Programming* (NLP) and even psychoanalysis. All of these have already been considered to be effective for PTSD and SUD pathologies.

Up to now, it was not clear by which mechanisms these therapies were able to treat patients. The integration concept allows us to propose that all of them may act in the same way, which is to introduce a reduced emotional response within the pathological memory, thereby reducing its disruptive consequences. All these therapies for psychiatric disorders, based upon reactivation-dependent memory malleability, are simple, inexpensive, and easy to arrange. Since these approaches have already provided promising results, they should be considered more seriously for clinical application in the near future for a number of other pathologies, such as phobias, feeding disorders, anxiety, etc.

By questioning the interpretation of numerous well-established aspects of memory processes, the integration concept adds to our understanding of the dynamic and flexible aspects of memory and by doing so opens new research approaches to treating various psychopathologies.

## Data Availability

All datasets generated for this study are included in the manuscript.

## Author Contributions

PG-V and CL wrote the article and approved it for publication.

## Conflict of Interest Statement

The authors declare that the research was conducted in the absence of any commercial or financial relationships that could be construed as a potential conflict of interest.

## Footnotes

^1^Amnesic treatments are always severe treatments which deeply affect the internal state, even when delivered within specific brain structures (Gisquet-Verrier et al., 2015). Accordingly, the internal state is an important aspect of the memory, the absence of which can induce retrieval difficulties.
